# Cutting a Long Story Short? The Clinical Relevance of Asking Parents, Nurses, and Young Children Themselves to Identify Children's Mental Health Problems by One or Two Questions

**DOI:** 10.1155/2014/286939

**Published:** 2014-12-31

**Authors:** Anne-Mari Borg, Raili Salmelin, Matti Joukamaa, Tuula Tamminen

**Affiliations:** ^1^Department of Child Psychiatry, School of Medicine, University of Tampere, 33014 Tampere, Finland; ^2^Department of Child Psychiatry, Tampere University Hospital, PL 2000, 33521 Tampere, Finland; ^3^School of Health Sciences, University of Tampere, 33014 Tampere, Finland; ^4^Department of Psychiatry, Tampere University Hospital, PL 2000, 33521 Tampere, Finland

## Abstract

*Background and Aims*. Assessing young children's mental health is a crucial and challenging task. The aim of the study was to evaluate the clinical relevance of asking parents, nurses, and young children themselves to identify children's mental health problems by only one or two questions. *Methods*. In regular health check-ups of 4- to 9-year-old children (*n* = 2682), parents and public health nurses assessed by one question whether the child had any emotional or behavioral difficulties. The child completed a self-evaluation enquiry on his/her emotional well-being. A stratified proportion of the participating parents were invited to a diagnostic interview. *Results*. Sensitivities were fairly good for the parents' (68%), nurses' (65%), and their combined (79%) one-question screens. Difficulties identified by parents and nurses were major risks (OR 10–14) for any child psychiatric disorders (*P* < 0.001). The child's self-evaluation was related to 2-fold to 3-fold risks (*P* < 0.05) for any psychiatric diagnosis, for any emotional diagnosis, and for negative situational factors. *Conclusion*. The one-question screen for parents and public health nurses together quite adequately identified the young children with mental health problems. The child's self-evaluation provided relevant and complementary information on his/her mental health and especially emotional problems.

## 1. Introduction

Assessing young children's mental health is a challenging task in primary services. Children's mental health problems are a global burden [1, 2] but, in general, their comprehensive screening is still in its infancy. It is necessary to develop and document validated and appropriate methods of screening for children's early mental health problems.

There are many special challenges in evaluating young children's mental health. Firstly, it is important to anchor the child's socioemotional and behavioural problems within the context of the child's developmental level [3]. Front line workers and parents may find it difficult to identify the child's psychopathology from the typical course of psychosocial development. Secondly, the well-being of the child is dependent on her/his family support and it cannot be evaluated in isolation from the well-being of the family. In addition to parents' reports, information on the child's symptoms and impairment in other significant social environments is needed [4]. Thirdly, interpretation and integrating all of the multi-informant and multimethod data is difficult and time consuming.

Every informant's evaluations count because no single informant's ratings can be used as “a gold standard” by which to measure psychopathology in children [5]. Discrepancies are common in different informants' ratings of child psychopathology [5]. Children's behaviour is known to be context dependent and parents' and teachers' reports are usually assessed. However, young children are rarely asked to self-evaluate their well-being.

Standardized self-report questionnaires are usually validated for school-aged children over 11 years old [6–8]. Young children's ability to provide reliable and useful information on their moods and feelings has been questioned. Evidence suggests that any agreement between the child's and adults' reports is stronger with respect to externalizing than to internalizing problems [4]. In addition, children tend to report more emotional symptoms than do their parents or teachers [4, 9–11]. It has recently been concluded that using only parental reports for assessing children's emotions results in an underestimation of emotional problems [11]. Thus it seems necessary to further develop and assess self-report methods also for young children.

Standardized screening questionnaires for children's mental health problems have been developed and some of them are well documented [6, 12, 13]. The number of screening tools available for assessing social-emotional functioning in the infant-toddler period and in preschool-aged children has also grown [3]. However, standardized questionnaires are seldom used regularly and comprehensively in monitoring children's mental health [14, 15]. Instead, asking ordinary questions of “How are you, how do you feel?” or “Do you have some difficulties or concerns?” seems to be the prevalent practices among health care professionals. Yet there is little evidence on how reliable and valid such ordinary concern questions are in identifying the children at risk for mental health problems.

Asking parents and teachers very shortly, by only one or a few questions, about their perceptions of the child's behavioural and emotional difficulties has been proven useful in recognising the children with mental health problems [13, 16, 17]. Ford et al. (2005) have found high values of specificity and negative predictive power for parental concerns evaluated by four questions, in screening child psychiatric disorders [16]. In that study, about half of the children of whom the parents reported at least one problem had a psychiatric disorder. The Strengths and Difficulties Questionnaire (SDQ) is a widely used short questionnaire in assessing children's mental health [12, 13]. In the first question on the SDQ impact supplement, the respondents are asked to evaluate whether the child has difficulties in one or more of the following areas: emotions, concentration, behaviour, or being able to get on with other people [18]. Notably, this one question has discriminated between community and clinical samples almost as well as the whole SDQ measure, and it has also predicted child psychiatric diagnosis quite accurately [13, 17].

Screening cost effectively for early problems in large groups of children necessitates multistage screening procedures [3]. In the present study, the focus of interest was on developing and testing as brief, simple, and easy-to-use a first-stage screening assessment tool as possible to identify children at elevated risk for mental health problems. The specific aims of the present study were

1. to assess the reliability and validity of a one-question screen presented to parents and public health nurses in everyday clinical practice in identifying children suffering from mental health problems,
2. to assess the clinical relevance of directly asking a young child to evaluate his/her emotional well-being.

## 2. Methods

### 2.1. Study Design

The study was a part of a project called“*Developing Children's Mental Health Work*, 2007–2009,” conducted in two hospital districts in Finland from March 2008 to March 2009. Altogether 154 child health clinics and school health care clinics participated in 25 municipalities. The respective local ethics committees approved the study. Informed consent was obtained from all participating parents.

Public health nurses introduced the study to parents making appointments for their 4−9-year-old children's regular health check-up. Prior to the visit to the clinic the study information and questionnaires were sent to interested parents at home: an informed consent form and a sociodemographic questionnaire including a parent's one-question screen and Strengths and Difficulties Questionnaires (SDQ) for both parents. The participating parents also asked the child's teacher in preschool education or at school to complete the SDQ. The parents returned all these forms to the public health nurse when attending the check-up. The design and sample of this phase of the study have been described in more detail elsewhere [19].

During the health check-up the child completed a self-evaluation enquiry about his/her well-being with the help of the public health nurse. In addition, the public health nurses completed a nurse's one-question screen for every child having a health check-up. After the check-up visit a feedback questionnaire on the feasibility of the child's self-evaluation enquiry was completed anonymously by the participating parents and once by each public health nurse involved in the process.

A subgroup of the participating parents was invited to a Development and Well-Being Assessment (DAWBA) interview. The SDQs were used to divide the children into screen-positive (scoring at or above the British 80th percentile cutoff, according to any informant) and screen-negative (scoring below the British 80th percentile cutoff, according to every informant) subgroups after the check-up visit. Every parent of a screen-positive child was invited to the DAWBA interview. For every two screen-positive cases (at the beginning of the study for every such case) a parent of a screen-negative child, matched for child's age group and gender, was invited to the DAWBA. With the parent's permission the child's teacher was also asked to complete DAWBA as a questionnaire. The interview phase of the study has been described in more detail elsewhere [20].

### 2.2. Sample

The sample consisted of 4- to 6-year-old preschoolers in child health clinics and 7- to 9-year-old children in school health care. Families not speaking Finnish were excluded from the study. Altogether 4,178 eligible children (49.5% girls) and their parents were invited to participate in the study, 3/5 of them being preschoolers (*n* = 2,596), the rest being school-aged (*n* = 1,582). The participation rate in the total sample was 64.2% (*n* = 2,682).

The participating parents filled in the parent's one-question screen in 98.9% of the cases. Of the 2,682 participating children 97.8% completed the self-evaluation enquiry. Both of these enquiries were available for 96.8% (*n* = 2,595) of the participating children. The public health nurses returned the nurse's one-question screen for 99.3% of participants.

Altogether 646 parental DAWBA interviews were available. Of these participants 67% were preschool-aged and 66% were boys. A teacher's report was available for 75% (*n* = 486) of the DAWBA participants.

Fifty-five percent of the participating parents and 68% of the public health nurses involved in the process (154/225) completed the feedback questionnaire on the feasibility of the child's self-evaluation enquiry.

### 2.3. Measures

The SDQ is a screening questionnaire for 4- to 16-year-olds to be completed by parents, teachers, and by 11- to 16-year-old children themselves [7, 12, 13]. In this study the Finnish version of the method, including both the symptom questionnaire and the impact supplement, was collected [18].

The SDQ symptom questionnaire consists of 25 items forming five subscales: emotional symptoms, conduct problems, hyperactivity/inattention difficulties, peer relationship problems, and prosocial behaviour. The items are scored as 1 for “somewhat true” and, depending on the item, as 0 or 2 for “not true” or “certainly true” and for analysis they were recoded as 0 to 2 for increasing severity. The scores from all the subscales except for the prosocial scale are summed to a total difficulties score in the range 0–40. Goodman [7] has proposed the 80th and 90th percentiles as provisional cutoffs for “borderline” and “abnormal.”

The first question on the parent and teacher version of the SDQ impact supplement asks, “Overall, do you think that your child/this child has difficulties in one or more of the following areas: emotions, concentration, behaviour or being able to get on with other people?” The answering alternatives are No, Yes—minor difficulties, Yes—definite difficulties, and Yes—severe difficulties. The rest of the impact supplement questions enquire, if difficulties are reported, about the duration or chronicity of the difficulties, overall distress, social impairment, and burden to others. The first question was used in the analyses of the present study. Otherwise, the reliability and validity properties of the extended version of the SDQ in the sample of Finnish 4–9-year-old children have been represented elsewhere [19, 20].

In the parent's one-question screen, parents were asked to assess whether their child had any emotional problems or any difficulties in behaviour, concentration, or social skills. The enquiry was answered on a four-step scale (no difficulties, not many difficulties, quite many difficulties, and very many difficulties). The enquiry was slightly modified from the first question on the parent's SDQ impact supplement.

In the nurse's one-question screen, public health nurses assessed, based on clinical evaluation, whether the child had, overall, difficulties in one or more of the following areas: emotions, behaviour, concentration, or being able to get on with other people. This was consistent with the first question in the parent's and teacher's SDQ impact supplement. The enquiry was answered on a five-step scale (no, yes/minor difficulties, yes/definite difficulties, yes/severe difficulties, and cannot say). The last answering option was added to the original alternatives of the abovementioned question on the SDQ.

**Figure 1:**
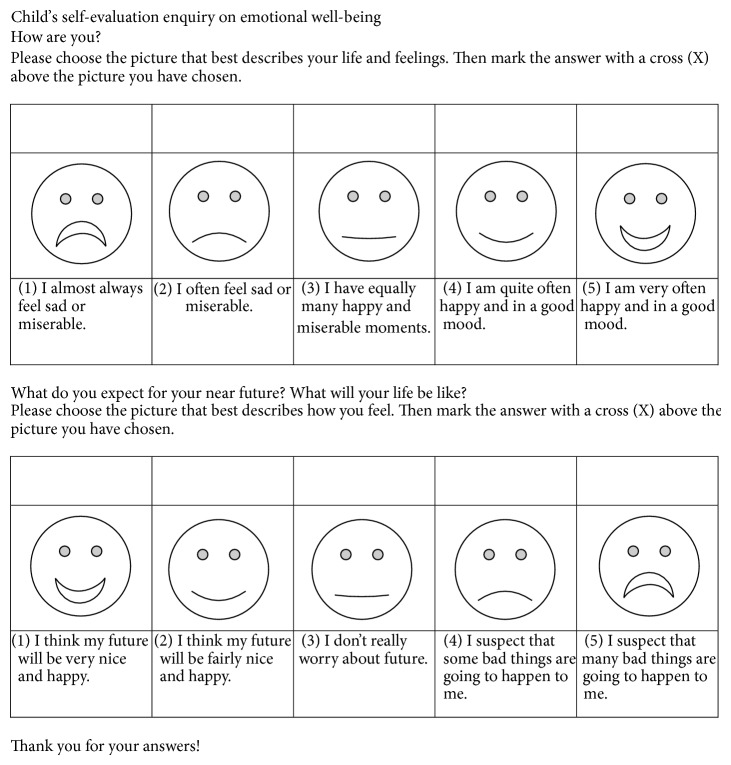
Child's self-evaluation enquiry on emotional well-being.

*The child's self-evaluation enquiry on emotional well-being* was developed for this study and consisted of two questions; see Figure 1. The written response alternatives had visual analogues in the form of facial expressions. The public health nurse read the questions and response alternatives to the child even if he/she could read. The child chose and marked with a cross the answer best describing his/her feelings.

The* DAWBA* method [21] consists of a semistructured interview, which can be administered to the parents of children aged 5 to 17 and to children over 11 years themselves; there is also a briefer questionnaire version for teachers. The structured questions cover most child psychiatric disorders and closely follow the diagnostic criteria according to the ICD-10 and DSM-IV. If definite symptoms are identified, parents are asked to describe the problems in more detail.

According to the responses of all available informants on the structured questions, the DAWBA program assigns each child to a level of an ordinal-scale measure which represents the prevalence of any diagnosis in epidemiological samples [22]. The categorization of this predictive measure offered to the clinical rater is <1% (very low), <5% (low), ≥20% (moderate), and ≥75% (high) [23]. To decide on definitive diagnoses a clinical rater then reviews all relevant information: the structured, closed, and open accounts of all available informants and the computer-predicted level of prevalence of any diagnosis.

The first author reviewed all the interviews and assigned the diagnoses according to ICD-10. The diagnoses were placed in five categories: emotional, conduct, hyperactivity, and other diagnoses (Tic/Tourette, pervasive developmental disorders, and not otherwise specified mental disorders) and situational factors (Z61 problems related to negative life events in childhood, Z62 other problems related to upbringing, and Z63 other problems related to primary support group, including family circumstances). The rater was trained by practising with the cases in the training manual [23] and participating in a two-day training course. When the diagnoses were uncertain, a consensus diagnosis was obtained by a consultation group of four experienced child psychiatrists. The frequency of diagnoses set by the rater was compared with the computer-predicted level of prevalence of any diagnosis. The associations were statistically highly significant (*P* < 0.001) between all pairs of the following groups: in the low prevalence group (<5%) 3% of the children were assigned to diagnoses, in the moderate prevalence group (≥20%) 38%, and in the high prevalence group (≥75%) 93% of the children.

In the* feedback questionnaire* on the feasibility of the child's self-evaluation enquiry, parents and public health nurses were asked how appropriate this method was in assessing the psychosocial well-being of the child (very good/fairly good/not good, not poor/rather poor/very poor). In addition, the public health nurses were asked to report how long, on average, to the nearest five minutes, it took to complete the child's self-evaluation enquiry and how burdensome they found it (not at all/not very/rather/very burdensome).

### 2.4. Statistical Analyses

The distributions of the multicategory parent's and nurse's one-question screens and the child's self-evaluation questions are expressed as percentages, and cross-informant agreements between them were examined with the *γ* coefficient. Because of the requirements of further analysis (examination of validity properties and logistic regressions) these questions were dichotomised in such a way that the upper category would include children with the strongest concerns and still be large enough for the analysis (see Table 1). Consequently, the categories were as follows: the parent's one-question screen, no/not many difficulties versus quite many/very many difficulties; the nurse's one-question screen, no/minor difficulties versus definite/severe difficulties; the first question of the child's self-evaluation enquiry (How are you?), very/quite often happy/as many happy as miserable moments versus often/almost always sad; and the second question (What do you expect?), very/fairly nice and happy future, do not really worry versus some bad/many bad things are going to happen to me. Furthermore, the answers of the two child's self-evaluation questions were combined as positive in both questions versus other combinations.

The DAWBA computer-predicted level of any child psychiatric diagnosis, dichotomised, according to the same principles as above, as <75% versus ≥75% (high prevalence level), was used as the gold standard in assessing the validity properties (sensitivity, specificity, and positive and negative predictive values [PPV, NPV]) of the abovementioned one-question screen for the parent, nurse, and child. All relevant two-variable analyses were conducted by comparing both age and gender groups or stratifying by them.

The DAWBA variable and the existence of any or selected specific diagnoses assigned by the rater were, one at a time, used as outcome variables in a set of logistic regression analyses. In the first set the only explanatory variable was one of the one-question screen variables (parent, nurse, and child) at a time, and the enter-method used thus produced unadjusted odds ratios (OR) for them. In the second set of logistic regression analyses, the explanatory variables comprised all one-question screens, gender, age group, and their interaction. To determine the strongest factors affecting the respective outcome variable, backwards stepwise method was used.

*P* values < 0.05 are considered to show statistical significance. The statistical analyses were accomplished with SPSS v. 19.

## 3. Results

### 3.1. Distributions of Parents' and Nurses' Perceptions and Children's Self-Evaluations

Table 1 shows the distributions of parents' and nurses' perceptions of the child's difficulties and children's self-evaluations in the total sample, stratified by gender and age groups. Six to seven percent of the children were evaluated by both the parents and the public health nurses to have definite or severe difficulties. According to both sets of informants' reports the proportion of boys having such difficulties was at least twice that of girls (*P* < 0.001) in the total sample and in both age groups. In addition, in parent's evaluations, the school-aged children were evaluated to have more commonly difficulties (8.5%) than the preschool-aged children (5.3%). The public health nurses could not say or did not know about the child's situation in 3.0% (*n* = 80) of the cases.

Of the children 2.1% evaluated themselves as feeling often or almost always sad or miserable (Table 1). Boys reported such negative feelings twice as commonly as girls (*P* < 0.001). Boys also reported more commonly than girls having “as many happy and miserable moments.” In the second question, 4.8% of children expected some or many bad things to happen. Younger children reported more commonly than older children negative feelings and future expectations.

*Cross-informant agreement* between the parents' and nurses' perceptions was fairly good (*γ* = 0.73) in the total sample. The agreements between child's self-evaluation and adults' evaluations were very low (child-parent *γ* = 0.10 and child-nurse *γ* = 0.15).

The agreement between the parent-rated one-question screen and the first question on the SDQ impact supplement was *γ* = 0.92.

### 3.2. Validity of the One-Question Screen against the Diagnostic Assessment

The sensitivity of the dichotomized parent's and nurse's one-question screen against the DAWBA computer-predicted high prevalence level of any diagnosis was fairly good (68% and 65%, resp., Table 2). The respective specificities were high (87-88%). PPVs were low and NPVs high. The sensitivity and PPV of the child's self-evaluation enquiries were very low (7–26%) and the specificity and NPV high (89–98%).

The sensitivities of the adult informants' perceptions were considerably higher and the specificities somewhat lower for boys than for girls (Table 2). In addition, the sensitivities were higher for older than for younger children. There were no differences in the values between the genders regarding the child's self-evaluation questions except in the second question, where the sensitivity for girls was higher than for boys (18% versus 6%). The PPV and NPV of the nurse's one-question screen and the child's self-evaluation questions were lower for boys than for girls, contrary to the results of the parent's responses.

Combining two or three of the informants' reports produced higher sensitivity than any of the respective single informants' reports. In the total sample, the sensitivity of the combined child's self-evaluation was 14% and the specificity was 93%. The sensitivity of both the combination of the parent's and nurse's perceptions and that of combining all three informants' reports was 79%, the respective specificities being 80% and 75%.

### 3.3. Risks for Child Psychiatric Disorders Related to the One-Question Screens

If parents or nurses identified difficulties the odds ratios for any and selected specific child psychiatric disorders were all statistically highly significant (*P* < 0.001, Table 3). The highest odds ratio (OR) related to difficulties identified by parents was that for a DAWBA computer-predicted high prevalence level (≥75%) of a child psychiatric diagnosis (OR 14.4), and the lowest (OR 4.5) was that for an emotional diagnosis. Nurse's assessment of definite or severe difficulties was most strongly associated with a hyperactivity diagnosis (OR 34.4) and least strongly with an emotional diagnosis (OR 4.0).

The negative rating in the combined child's self-evaluation was statistically significantly (*P* < 0.05) associated with a DAWBA computer-predicted high prevalence level of any diagnosis (OR 2.2), with any DAWBA-rater assigned diagnosis (OR 2.4), with any emotional diagnosis (OR 3.0), and with negative situational factors (OR 3.2).

Examining the effects of the evaluations of all three informants simultaneously by backwards stepwise logistic regression revealed that the difficulties identified by parents and nurses remained the strongest and significant risk factors for all child outcomes (OR 2.7–7.1), except in predicting a hyperactivity diagnosis, where only difficulties identified by the nurses remained statistically significant (OR 20.9); see Table 4. The child's self-evaluation remained a statistically significant risk factor for any emotional diagnosis (OR 2.7) and for negative situational factors (OR 2.9). Girls had higher risk than boys for any emotional diagnosis (OR 2.3) and school-aged children had higher risk than preschoolers for any assigned diagnoses (OR 1.8).

### 3.4. Feedback on the Child's Self-Evaluation Enquiry

The child's self-evaluation enquiry was considered to be very or fairly age-appropriate for assessing the child's psychosocial well-being by 63% of the parents and by 71% of the public health nurses in the total sample. Eight percent of the parents and 9% of the public health nurses evaluated the appropriateness of the enquiry to be rather or very poor. Fourteen percent of the parents had no opinion on the subject.

Most (96%) public health nurses found the two questions not very or not at all burdensome. Almost all (99%) the public health nurses completed the enquiry with the children in 10 minutes or less.

## 4. Discussion

The main results of the study suggested that the one-question screen presented to parents and public health nurses offers a valid and clinically relevant guide in identifying children suffering from mental health problems. It is also useful to hear the young child's own perspective when trying to identify children at high risk, especially for emotional problems.

Of the children having regular health check-ups 6-7% were evaluated by the parents or the public health nurses in this study to have definite or severe difficulties. In a British epidemiological sample 9.5% of parents reported concerns about their child's emotions, behaviour, or activity level [24]. The present finding compares closely with earlier 5–24% frequencies of psychiatric symptoms or disorders in population samples of young children [20, 25–28]. As in some earlier studies boys were more commonly than girls reported to have difficulties [25, 28, 29] or any disorder [28]. The present finding that older children had more parent-rated difficulties than younger children should be considered preliminary. The earlier findings on differences between the score distributions split by comparable age groups have been inconsistent according to the computerised multicultural norms of the SDQ [18].

In the present study only 2% of children evaluated themselves as feeling often or almost always sad or miserable. The child's self-reported frequency of emotional problems in this study was lower than in earlier studies with validated assessment methods [30, 31]. Recently, 12–16% of Belgian 5- to 10-year-old children reported emotional problems, such as anger, anxiety, and sadness, in a short self-report questionnaire [11]. Further studies are needed on young children's self-reported frequencies of emotional problems in community and clinic samples.

The cross-informant agreement between the parent's and public health nurse's perceptions was fairly good but the agreements between child's self-evaluation and adults' evaluations were very low. In the present study, it was not our purpose to compare the child's and the adults' reports because adult informants answered a similar question on overall difficulties whereas the child's questions focused on his/her emotional well-being and expectations. However, the weak correlation between the parent's and the child's evaluations was in concordance with earlier studies [4, 10, 11]. As expected, the agreement between the parent's one-question screen and the first question on the SDQ impact supplement was high. The present one-question screen was only slightly modified from the original abovementioned question on the SDQ.

The single question for the parent and public health nurse had an adequate capacity to discriminate between the low-risk and high-risk children in the sample. The child's self-evaluation, however, was not sensitive for identifying high-risk children. The one-question screen for the parent and public health nurse detected two-thirds of the children with a psychiatric disorder and the specificity, the proportion of true negatives, of the adults' evaluations was high. Of the children identified as having difficulties, 41% had a computer-predicted DAWBA diagnosis and of those children identified as having no difficulties only 5% had a respective diagnosis. Thus the parent's and public health nurse's perceptions of difficulties were found to be fairly good and evaluations of no concern about the child's situation were quite accurate.

The present values of sensitivity and specificity for the one-question screen for parents concur closely with earlier results on the validity values of screening questionnaires [6, 12, 32]. We replicated the earlier finding that a single question on whether the child has emotional or behavioural difficulties discriminates almost as well as a whole questionnaire comprising many items between low-risk and high-risk children [13]. Further information was also gained on the effect of combining two or three different informants' answers, which was found to produce higher sensitivity values compared to a single informant's report. The parents and nurses together identified four-fifths of the children with a psychiatric diagnosis. Obviously, the combined parent's and public health nurse's one-question screen seemed to be a good indication for a more comprehensive evaluation of the child's mental health.

Difficulties identified by parents and nurses were found to be strong and statistically significant risk factors for any child psychiatric disorders. The highest odds ratio for parental perception of difficulties was found for any child psychiatric diagnosis (OR 14.4) and for public health nurse's respective perception for a hyperactivity diagnosis (OR 34.4). Both informants' concerns had the lowest OR for an emotional diagnosis, being still a fourfold to fivefold risk. Difficulties identified by parents and nurses remained the strongest risk factors for most of the child's outcomes when all the predictors, including child's age and gender, were taken into account. The present strong association between difficulties identified by parents and a child's psychiatric diagnosis is comparable to the earlier finding of a strong association (OR 16) between high scores on the SDQ parent report and a child psychiatric disorder [12]. The present results suggest that the one-question screen for parents and public health nurses yields validated and supplementary information on the child's risks for mental disorders.

When the young children reported low mood or negative expectations this was related (*P* < 0.05) to elevated risks for a psychiatric disorder, emotional disorders, and negative situational factors in the family. When taking all the risk factors into account, the child's self-evaluation remained as a statistically significant threefold risk for any emotional diagnosis and for negative situational factors. For an emotional diagnosis girls were found to have a twofold risk compared to boys. Older children had a twofold risk for any assigned diagnoses compared to younger children. The present findings confirmed that the young child's self-evaluation yields relevant and complementary information on the child's emotional well-being from the child's inner perspective.

Although the screening properties of the one-question screen were quite adequate, one-fifth of the children with a child psychiatric disorder were not identified by their parents or public health nurses. The clinicians should remember that even if they use a standardized screening method there will remain a proportion of these “false negative” children. It is a special challenge to try to identify these children in need of psychosocial support. In addition, whatever screening method was used it needs to be administered systematically in order to produce reliable results.

Several limitations should be noted in the study. The child's self-evaluation enquiry had not been tested before, and therefore further studies are needed on the psychometric screening properties of similar brief screening assessments for children. Possible effects of the moderate participation rates in the first phase and in the interview phase of the study as well as limitations related to diagnostic procedures have been discussed elsewhere [19, 20].

The strengths of the study were the multi-informant approach and the large sample of young children. The study was conducted in an everyday clinical setting of children's regular health check-ups, thus improving the usability of the results. The discriminative validity properties of the single questions were assessed against a diagnostic assessment as a gold standard. This made it possible to explore the child's symptoms and level of impairment in a multi-informant approach and in different contexts, also including the child's teacher's report. The study presents further information about the very brief screening assessments for parents and public health nurses.

The present study generated new information about directly asking a young child to evaluate his/her emotional well-being by two pictorial questions in a clinical setting. We found no earlier corresponding studies. Some pictorial self-report questionnaires for preschool and for young schoolchildren are available [4, 33–35]. The correspondence between self-reports of children and the reports of parents and teachers was not altered when pictorial self-report questionnaires were used instead of traditional verbal self-report instruments [4]. The use of pictures combined with verbal questions, however, was assumed to help children in communicating their opinions in the present study. The parents and public health nurses gave positive feedback on the feasibility of the child's self-evaluation enquiry in the context of regular health check-ups.

## 5. Conclusions

The results suggest that the one-question screen for parents and public health nurses together adequately identifies those young children with mental health problems and can thus be considered as a first step screening assessment in everyday clinical front-line practice. In addition, the young child's self-evaluation questions yielded complementary and relevant information on their mental health and especially emotional problems, speaking for the importance of directly asking the child's own perspective.

## Figures and Tables

**Table 1:** Distributions of answers in the parents' and nurses' one-question screen and child's self-evaluation in a community sample of Finnish 4–9-year-old children.

	Total sample			χ^2^	Preschoolers			School-aged children			χ^2^
	All	Boys	Girls	df^1^	All	Boys	Girls	All	Boys	Girls	df^1^
	%	%	%	P^ 2^	%	%	%	%	%	%	P^ 3^
Parent's one-question screen	(n = 2652)	(n = 1295)	(n = 1357)	80.79	(n = 1757)	(n = 867)	(n = 890)	(n = 895)	(n = 428)	(n = 467)	13.88
No difficulties	47.0	38.7	55.0	2	46.3	38.2	51.2	48.5	39.7	56.5	2
Not many difficulties	46.6	52.4	41.0	<0.001	48.4	54.4	42.6	43.0	48.4	30.1	0.001
Quite many/very many difficulties	6.4	8.9	4.0		5.3	7.4	3.3	8.5	11.9	5.4	
Nurse's one-question screen^4^	(n = 2602)	(n = 1269)	(n = 1333)	81.73	(n = 1722)	(n = 847)	(n = 875)	(n = 880)	(n = 422)	(n = 458)	0.37
No difficulties	75.5	67.8	82.8	2	75.2	68.5	81.7	76.1	66.6	84.9	2
Yes—minor difficulties	17.7	22.5	13.1	<0.001	18.0	22.1	14.1	17.0	23.2	11.4	ns.
Yes—definite/severe difficulties	6.8	9.7	4.1		6.8	9.4	4.2	6.8	10.2	3.7	
Child's self-evaluation											
How are you?	(n = 2623)	(n = 1287)	(n = 1336)	22.86	(n = 1739)	(n = 860)	(n = 879)	(n = 884)	(n = 427)	(n = 457)	446.33
Very often happy	54.0	51.4	56.4	3	68.0	66.3	69.6	26.5	21.5	31.1	3
Quite often happy	28.9	28.0	29.7	<0.001	18.5	17.1	19.8	49.3	49.9	48.8	<0.001
Equally many happy and lousy moments	15.1	17.7	12.5		10.9	13.0	8.9	23.2	27.2	19.5	
Often/almost always sad	2.1	2.9	1.3		2.6	3.6	1.7	1.0	1.4	0.7	
What do you expect for your near future?	(n = 2620)	(n = 1284)	(n = 1336)	10.16	(n = 1737)	(n = 858)	(n = 879)	(n = 883)	(n = 426)	(n = 457)	115.01
Very nice and happy days	50.1	48.5	51.6	3	55.8	55.4	56.3	38.8	34.7	42.7	3
Quite nice and happy days	29.4	28.8	30.0	<0.017	23.0	22.3	23.8	42.0	42.0	42.0	<0.001
Not bothering	15.7	18.0	13.5		15.4	17.0	13.9	16.2	20.0	12.7	
Some/many bad things are going to happen	4.8	4.7	4.9		5.7	5.4	6.0	2.9	3.3	2.6	

^1^df: degrees of freedom.

^2^Tested between genders.

^3^Tested between age groups.

^4^Answering alternatives “do not know” and “cannot say” extracted from analyses (*n* = 80).

**Table 2:** Sensitivity, specificity, and positive (PPV) and negative (NPV) predictive values of the parent's and nurse's one-question screen and child's self-evaluation questions (no or mild concerns/problems versus more severe options) calculated against the DAWBA computer-predicted prevalence level of any diagnosis (<75% versus ≥75%) in a sample of Finnish 4–9-year-old children.

	High prevalence level of diagnosis																			
	Total sample				Boys				Girls				Preschoolers				School-aged children			
	Sens^1^	Spec^2^	PPV^3^	NPV^4^	Sens^1^	Spec^2^	PPV^3^	NPV^4^	Sens^1^	Spec^2^	PPV^3^	NPV^4^	Sens^1^	Spec^2^	PPV^3^	NPV^4^	Sens^1^	Spec^2^	PPV^3^	NPV^4^
	%	%	%	%	%	%	%	%	%	%	%	%	%	%	%	%	%	%	%	%
Parent's one-question screen	(*n* = 637)				(*n* = 423)				(*n* = 214)				(*n* = 424)				(*n* = 213)			
	68	87	41	95	73	86	45	96	50	89	30	95	70	90	44	96	65	82	39	93
Nurse's one-question screen	(*n* = 622)				(*n* = 411)				(*n* = 211)				(*n* = 414)				(*n* = 208)			
	65	88	41	95	69	85	42	95	56	92	40	96	72	88	42	97	55	87	40	92
Child's self-evaluation																				
How are you?	(*n* = 629)				(*n* = 418)				(*n* = 211)				(*n* = 418)				(*n* = 211)			
	7	98	26	89	8	97	25	88	6	99	33	92	10	97	29	91	3	98	20	86
What do you expect for your near future?	(*n* = 628)				(*n* = 417)				(*n* = 211)				(*n* = 418)				(*n* = 210)			
	9	94	16	89	6	95	14	87	18	93	19	93	10	94	15	91	7	96	20	86
Combined Child's self-evaluation	(*n* = 629)				(*n* = 418)				(*n* = 211)				(*n* = 418)				(*n* = 211)			
	14	93	20	90	13	93	21	88	18	93	19	93	18	92	19	91	10	95	23	86
Combined parent's and nurse's one-question screen	(*n* = 613)				(*n* = 405)				(*n* = 208)				(*n* = 407)				(*n* = 206)			
	79	80	34	97	85	77	36	97	61	84	27	96	84	82	35	98	72	75	32	94

^1^Sensitivity; ^2^specificity; ^3^positive predictive value; ^4^negative predictive value.

**Table 3:** The odds ratios (OR) for child outcomes related to parent's and nurse's evaluation of the child's difficulties and child's self-evaluation of emotional well-being according to DAWBA assessment in a sample of Finnish 4–9-year-old children (*n* = 646). The OR of each separate evaluation (no or mild difficulties/concerns versus more severe options) for each outcome measure is shown.

	Computer-predicted prevalence^1^	Rater-assigned child psychiatric ICD-10 diagnosis					
		Any	Emotional	Conduct	Hyperactivity	Other^2^	Situational factors^3^
	OR (95% CI)	OR (95% CI)	OR (95% CI)	OR (95% CI)	OR (95% CI)	OR (95% CI)	OR (95% CI)
Parent's concern enquiry	14.4^**^ (8.4–24.9)	9.9^**^ (6.3–15.6)	4.5^**^ (2.6–19.2)	9.9^**^ (5.1–18.9)	8.1^**^ (3.9–16.8)	9.7^**^ (4.0–23.2)	7.3^**^ (3.8–14.0)
Nurse's concern enquiry	13.6^**^ (7.8–23.5)	12.4^**^ (7.8–19.7)	4.0^**^ (2.2–7.1)	10.0^**^ (5.2–19.3)	34.3^**^ (12.9–91.1)	5.8^**^ (2.5–13.3)	8.2^**^ (4.2–16.0)
Child's self-evaluation (two questions combined)	2.2^*^ (1.1–4.7)	2.4^*^ (1.3–4.5)	3.0^*^ (1.4–6.5)	2.0 (0.85–5.0)	1.2 (0.4–4.2)	1.1 (0.3–5.0)	3.2^*^ (1.4–7.5)

^1^Prevalence level <75%/≥75%.

^2^Tic/Tourette, pervasive developmental disorders, and not otherwise specified mental disorders.

^3^Factors influencing health status and contact with health services (ICD-10): Z61 problems related to negative life events in childhood, Z62 other problems related to upbringing, and Z63 other problems related to primary support group, including family circumstances.

^*^ *P* < 0.05.

^**^ *P* < 0.001.

**Table 4:** The odds ratios (OR) for child outcomes related to the combined effects of the parent's and nurse's one-question screen and the child's self-evaluation of emotional well-being as well as child's gender and age group. The OR for the variables remaining in the model at the last step of each backwards stepwise logistic regression are shown.

Variables entered into each model	Computer-predicted prevalence^1^ (*n* = 68)	Rater-assigned child psychiatric ICD-10 diagnosis					
		Any (*n* = 117)	Emotional (*n* = 53)	Conduct (*n* = 41)	Hyperactivity (*n* = 32)	Other^2^ (*n* = 23)	Situational factors^3^ (*n* = 38)
	OR (95% CI)	OR (95% CI)	OR (95% CI)	OR (95% CI)	OR (95% CI)	OR (95% CI)	OR (95% CI)
Parent's one-question screen^4^	6.7^**^ (3.6–12.7)	4.3^**^ (2.5–7.4)	2.7^*^ (1.3–5.5)	4.4^*^ (2.0–9.6)	2.1 (0.9–5.1)	4.7^*^ (1.7–13.0)	4.0^*^ (1.8–8.9)
Nurse's one-question screen^4^	6.6^**^ (3.5–12.5)	7.1^**^ (4.1–12.1)	2.9^*^ (1.4–5.9)	4.9^**^ (2.3–10.7)	20.9^**^ (7.2–60.4)	3.1^*^ (1.1–8.4)	3.5^*^ (1.6–7.7)
Child's self-evaluation^4^ (combined)		2.1 (1.0–4.6)	2.7^*^ (1.2–6.2)				2.9^*^ (1.1–7.4)
Child's gender^5^			2.3^*^ (1.3–4.3)		0.4 (0.1–1.1)		
Child's age^6^		1.8^*^ (1.1–2.9)				2.5 (1.0–6.2)	
Gender ∗ age	—^7^					—^7^	

^1^Prevalence level <75%/≥75%.

^2^Tic/Tourette, pervasive developmental disorders, and not otherwise specified mental disorders.

^3^Factors influencing health status and contact with health services (ICD-10): Z61 problems related to negative life events in childhood, Z62 other problems related to upbringing, and Z63 other problems related to primary support group, including family circumstances.

^4^No or mild difficulties/concerns versus more severe options.

^5^Girls versus boys.

^6^School-aged versus preschool children.

^7^The variable remained in the model but OR could not be computed because there were too few cases in some of the subgroups.

^*^ *P* < 0.05.

^**^ *P* < 0.001.
